# Beef Tea

**Published:** 1874-06

**Authors:** 


### BEEF TEA

has but little of that kind of nourishment which forms flesh and gives strength, but it does contain potash, which is essential to building up the stamina of the system. It is particularly valuable when persons have been very low, or have been a long time sick; then, if there is even a slight relish for it, take a little at a time and often, and it will regenerate the organism rapidly. It is made thus: Lay a thin, lean steak on a board, scrape it with a knife until nothing is left but white fibres; mix what is scraped off, very thoroughly, in three times the amount of cold water; put it on the fire and let it come slowly to a boil, stirring it well all the time to prevent burning or caking. As soon as it comes to a boil, remove, cool it and season to taste. Take from two to six tablespoonfuls at a time. This, says *Health at Home*, is the best concentrated meat fibre, and the most nutritious beef tea that can be made.

